# Effect of the Interface/Interphase on the Water Ingress Properties of Joints with PBT-GF30 and Aluminum Substrates Using Silicone Adhesive

**DOI:** 10.3390/polym15040788

**Published:** 2023-02-04

**Authors:** Catarina S. P. Borges, Eduardo A. S. Marques, Ricardo J. C. Carbas, Alireza Akhavan-Safar, Christoph Ueffing, Philipp Weißgraeber, Lucas F. M. da Silva

**Affiliations:** 1 Instituto de Ciência e Inovação em Engenharia Mecânica e Engenharia Industrial (INEGI), 4200-465 Porto, Portugal; 2 Robert Bosch GmbH, Corporate Research and Advance Engineering, 71272 Renningen, Germany; 3 Chair of Lightweight Design, University of Rostock, 18051 Rostock, Germany; 4 Departamento de Engenharia Mecânica, Faculdade de Engenharia (FEUP), Universidade do Porto, 4200-465 Porto, Portugal

**Keywords:** adhesive joints, water uptake, interface, interphase, composite substrates, metallic substrates

## Abstract

The aim of this work is to analyze the difference between silicone/composite and silicone/metal interphases, both in terms of water diffusion behavior and failure of the aged joints. For that, silicone joints with two different suhbstrates were prepared. The substrates were polybutylene terephthalate with 30% of short glass fiber (PBT-GF30) and 6082-T6 aluminum. It is assumed that the water uptake of the joints is equal to the water uptake of the substrate, adhesive, and interphase. Therefore, knowing the first three, the last could be isolated. To study the water diffusion behavior of the complete joint, rectangular joints were prepared, immersed in water and their water uptake was measured. The water immersion was conducted at 70 °C. It was concluded that the aluminum/silicone joints absorbed more water through the interphase region than the PBT-GF30/silicone joints, since the difference between the expected water uptake and the experimentally measured mass gain is significantly higher, causing adhesive failure of the joint. The same was not observed in the PBT-GF30/silicone, with a more stable interphase, that does not absorb measurable quantities of water and always exhibits cohesive failure.

## 1. Introduction

Two components can be joined using different methods, such as riveting, bolting, or welding. However, on one hand, riveting and bolting rely on the insertion of holes, creating stress concentration sites, which is particularly concerning when joining composite substrates. On the other hand, welding heavily depends on the substrate material and requires the local heating of the components, which can introduce a region of degraded properties. Therefore, adhesive bonding in an interesting solution, allowing to bond both metals and composites, and even enabling the assembly of dissimilar materials [1,2,3,4].

One of the main setbacks of adhesive joints is their sensitivity to humid environments. Since, due to their polymeric matrix, they generally absorb water and it can highly influence their mechanical properties. Additionally, some adhesives, such as silicones, are often used to not only bond assemblies, but also to seal them. Therefore, it is important to understand the water uptake behavior of silicone adhesive joints to ensure they are following their sealing purpose. In adhesive joints, water may follow different paths, either through polymeric materials (such as the adhesive or the matrix of the substrate, if it is a composite material), through the pores and cracks of the adhesive or through the adhesive/substrate interface [5].

Considering the moisture absorbed by the bulk materials, it can be absorbed as free water or bound water. Free water fills the free spaces left in the polymeric chain of the material. Bound water creates chemical bonds [6,7]. The water ingress behavior of materials is not described by a single universal rule. However, in the majority of the cases, the Fick’s law can be used to fit the empirical results [8,9,10,11,12]. This law is defined by the coefficient of diffusion, *D*, which is related to the diffusion rate, and the water uptake at saturation, $M_{\infty }$. In the case of the family of adhesive used in this study, silicones, the Fick’s law of diffusion has been used by several authors. Dai et al. [13], for instance, used Fick’s diffusion law to fit the water uptake behavior of silicone exposed to deionized water at temperatures between 20 and 60 °C. As temperature increases, the coefficient of diffusion also increases, but the water uptake at saturation was described as fairly insensitive to temperature. Khalilullah et al. [14] also studied silicone rubbers and concluded that not only does the material follow a Fickian law of diffusion, but also that the Arrhenius law can be used to describe the influence of temperature and relative humidity on the increase in the diffusion coefficient of the material. The same conclusions were reached by Borges et al. [15], that studied the same silicone adhesive used in this work. However, there are authors that use different diffusion laws, such as Lutz et al. [16], that used the Langmuir model to describe the water diffusion on high temperature vulcanized silicone (HTV-SR), and Fang et al. [17]. Ali and Hackman [18] studying high-temperature vulcanized (HTV) silicone rubber, understood that, at a high temperature (98 °C), as immersion time increases, the silicone becomes increasingly less hydrophobic.

Adhesives exposed to water typically present a decrease in their mechanical properties, such as the elastic modulus [19,20,21] and strength [22,23]. The ductility is known to increase, leading to an increase in strain-to-failure and creep strain [20,22,24]. Regarding the toughness of the material, typically, decreases are reported [25,26,27]. As immersion temperature increases and the glass transition temperature is approached, since the water uptake is dependent on the microstructure and chemical bonds of the material, adhesives absorb more water [28] and, since aging decreases this temperature, it can lead to a higher water absorption even if the immersion temperature is not changed. However, silicones present a particular behavior. Fan et al. [29] exposed silicone to an environment of 55 °C and 85% of relative humidity (RH) for 42 days. The silicone was periodically removed from immersion and tested. Over the tested time period, the material increased its strength and stiffness. Borges et al. [15], studying the same material used in this work, concluded that water immersion does not significantly change the properties of the adhesive for immersions in a water container placed in a climatic chamber at temperatures between 70 and 130 °C.

Regarding the substrate, if they are metallic, they can oxidize or corrode. However, they are not expected to absorb water. Nonetheless, if working with composite substrates with polymeric matrices, the same effect recorded for bulk adhesives is expected. Borges et al. [30] studied the effect of moisture on the mechanical properties of one of the particular substrates used in the present study, polybutylene terephthalate with 30% of short glass fiber (PBT-GF30). This material also follows the Fick’s law of diffusion, increasing its water ingress rate with temperature, according to the Arrhenius law. Additionally, it was seen that, for immersion temperatures between 80 °C and 85 °C, chemical changes start occurring in the material. Brandão et al. [31] studied the effect of immersion temperature on single lap joints (SLJ) and double cantilever beam (DCB) joints manufactured from the same adhesive and substrate material used in this work. Immersion in water inside a climatic chamber in which the temperatures were set to 70 °C, 90 °C, and 130 °C was studied and the specimens were analyzed dried at the considered temperature, after water aging and redried. It was concluded that the aged samples had a higher failure load, due to the slight increase in strength of the silicone when aged, due to what seemed to be a post-curing effect. However, the DCB joints aged at 90 °C and both aged and redried in the climatic chamber at 130 °C exhibited failure in the substrate due to the chemical changes in the PBT-GF30 material.

Until this point, the effect of water on the bulk materials, adhesive, and substrates was discussed. However, as said, there are two additional paths of water diffusion, through the pores and cracks of the adhesive and through the interface. The latter is referred to, by several authors, as the main cause of degradation of the joint [32].

When two or more substrates are joined using an adhesive, there is the creation of a layer, between the adhesive and each substrate, that is the interphase. This zone is a layered region, which has properties different from those of the adhesive and substrate, and this makes the interphase a region of heterogeneity, with a gradient of mechanical, chemical, and thermal properties, among others [1,33]. Within the interphase, there is the plane of contact between adhesive and substrate, which is the interface [1]. In the case of metallic substrates, the oxides that can be found on the surface of the substrate may be dissolved in the adhesive layer close to the substrate. Considering one of the most commonly used adhesive systems, epoxy-amine, it has been seen that, on one hand, the amine is chemically adsorbed by the oxide on the substrate and, on the other hand, the amine component, which is basic, dissolves the oxide layer [33,34]. Ultimately, it has been observed a preferential adsorption of the curing agent, amine, by the substrate during the curing process of the adhesive, which can inhibit chemical reactions between epoxy and curing agent, due to excess of epoxy, paired with an overcuring of the epoxy near the substrate, due to the excess of amino groups [4,5,35]. The study of silicone adhesive joints is less common, and is one of the motives behind the relevance of this research.

When exposed to water, joints commonly show a higher water uptake than what would be expected if water was only absorbed through the adhesive. Zanni-Deffarges and Shanahan [25] analyzed the coefficient of diffusion of an epoxy adhesive in bulk and in an adhesive joint. It was reported that, in the adhesive joint, the coefficient of diffusion is almost one order of magnitude higher, validating that there are alternative paths of water diffusion. The path of water diffusion suggested was the interface between the adhesive and substrate where, due to capillarity diffusion, the water diffuses quicker than in the bulk adhesive. Cognard et al. [36] suggests that, when water is absorbed by materials with pores, it will condensate when its chemical potential is equal to that of the liquid. Therefore, in an adhesive joint there are pores close to the interface, the water condensates and agglomerates close to the interface, creating a debonding pressure. Combining these findings with the conclusions of Zanni-Deffarges and Shanahan [25], it can be inferred that, in adhesive joints, water diffuses quicker through the interface than through the bulk adhesive and promotes debonding. It is important to point out that these conclusions were drawn from adhesive joints with metallic substrates.

Drain et al. [37] analyzed polycarbonate (PC) and steel joints, bonded with a cyanoacrylate adhesive. It was concluded that, when bonding PC substrates, the interface could not be identified due to the dissolution of the composite in the adhesive, particularly in the ethylcyano monomer. However, for steel substrates, the interface was identifiable. This translates into a higher water resistance of the interface of the PC joint, when compared to that of the steel joint, because the water diffusion through the interface is negligible. Li et al. [38] studied the effect of hygrothermal aging on joints with dissimilar substrates, carbon fiber reinforced plastic (CFRP), and aluminum. It was concluded that the strength of the joint decreases with increasing aging time and failure moves from cohesive in the adhesive to interfacial at the adhesive/aluminum interface, moving again to the adhesive when aging time further increases. Franco et al. [39] analyzed the effect of surface preparation and concluded that when the aluminum is anodized using a tartaric–sulfuric acid-based process, the strength of the interface is maintained for longer immersion times, when compared to mechanical abrasion.

In many applications where the adhesive is subjected to high-temperature and humid environments, silicones are used, and not epoxies, since this material is usually used as sealant for its elastic properties and for its coefficient of thermal expansion, which allows to accommodate different thermal expansions of the materials being bonded. This work arises from the study of a housing for electronic components manufactured from PBT-GF30 and bonded using a silicone adhesive, to seal the assembly. The silicone adhesive is also used to bond the housing to a heat sink, manufactured from aluminum. As said, the bulk materials, adhesive and substrate, were already studied in terms of water diffusion behavior and its effect on the mechanical properties [15,30]. Additionally, the effect of moisture on SLJs and DCBs with PBT-GF30 substrates and the same silicone adhesive was analyzed. However, in this case, the effect of the interphase was not isolated [31].

As seen, there are studies analyzing the effect of interfaces on the water diffusion of adhesive joints with epoxy adhesives and metallic substrates, and some first approaches trying to compare metallic and composite substrates. At this point, a remark must be made between interfaces and interphases. Generally, when studying this problematic, the water uptake between the adhesive and substrate is attributed to the interface. However, in most cases, there is no clear micro or nanoscale research performed to ensure the path of water diffusion and failure occurs exactly between adhesive and substrate (interface), and not in the interphase region. Therefore, since the interphase comprehends the interface, these results can be generally attributed to the interphase without harming the main conclusions drawn.

The durability of silicone/metal (aluminum) and silicone/composite (PBT-GF30) joints was not yet studied in detail. The interphase water uptake behavior needs to be established and the effect on the mechanical properties studied. The main aim of this work is analyzing the effect of interfaces and, more generally, interphases, on the water diffusion behavior of joints with silicone adhesive and both aluminum and PBT-GF30 substrates, along with the influence of water absorption on the mechanical properties of the joints. The water diffusion analysis was fully performed at 70 °C. To isolate the water diffusion behavior of the interphase, for both substrate materials, rectangular joints (RJ) with 0.5, 1, and 2 mm of adhesive thickness were prepared. A gravimetric study was performed and, knowing the water diffusion behavior of the bulk materials at this temperature, determined in previous studies [15,30], the diffusion at the interphase was isolated. Afterwards, SLJs with the same adhesive thicknesses were prepared, and tested before water aging. The joints were immersed in water for the time needed to reach a constant mass and tested again, to analyze the effect of water on the mechanical properties and on the fracture surface of the joints.

## 2. Experimental Details

### 2.1. Methodology

**Figure 1:**
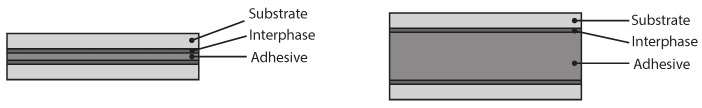
Different adhesive thicknesses to isolate the effect of the interphase, thin (**left**) and thick (**right**).

This work aims to isolate the effect of the interface on silicone/aluminum and silicone/ PBT-GF30 adhesive joints. Therefore, first, joints with three different adhesive thicknesses were prepared, since, knowing the adhesive and substrate behavior and assuming the thickness of the interphase insensitive to the thickness of the adhesive, its behavior can be isolated, Figure 1.

The same is valid if the water uptake is not globally through the interphase but specifically through the interface, if the chemical affinity of the adhesive and substrate is lower, since the contact plane is also the same regardless of adhesive thickness.

The joints were immersed in water and their water uptake recorded. Generally, for all joints, it can be said that the mass gain of the joint is the sum of the mass gain of the substrate, of the adhesive and of the interphase:

(1) $$\Delta m_{j}=\Delta m_{s}+\Delta m_{a}+\Delta m_{i}$$

where $\Delta m_{x}$ refers to the mass gain and *j* refers to the joint, *s* to the substrate, *a* to the adhesive and *i* to the interphase.

**Figure 2:**
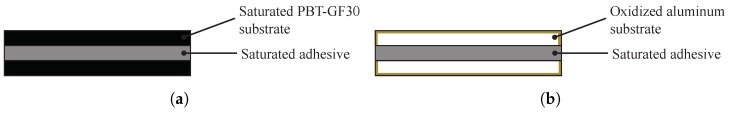
Effect of water on PBT-GF30 (**a**) and aluminum joints (**b**).

In the case of PBT-GF30 joints, the mass gains of the adhesive and the substrate occur due to water uptake, and are, therefore, volumetric phenomena, as shown in Figure 2a. For joints with aluminum substrates, the mass gain of the adhesive is due to water uptake, however, the mass gain of the substrate is due to the oxidized layer that is created at the surface, as shown in Figure 2b.

In the experimental gravimetric test, the mass of the joint is measured as a whole. However, the objective is to isolate the effect of the interphase. Therefore, it is necessary to eliminate the contribution of the adhesive and substrate from the equation. Considering that the real mass gain is the measured mass gain given by the combination of the factors described in Equation (1) and that the theoretical mass gain would be the mass gain in the case where only adhesive and substrate absorb water, the mass gain of the interphase would be given by:

(2) $$\begin{array}{c} \Delta m_{i}=\Delta m_{j(a+s+i)}^{R}-\Delta m_{a+s}^{T} \end{array}$$

where *R* and *T* refer to real and theoretical, respectively. Therefore, the concern now is calculating the theoretical mass gain of the joint.

#### 2.1.1. Joints with PBT-GF30 Substrates

The water uptake behavior of PBT-GF30 and silicone has already been determined in other work by the same authors [15,30]. Therefore, it is possible to conduct a numerical simulation that is going to predict the mass gain of the adhesive and substrate and, finally:

(3) $$\begin{array}{c} \Delta m_{a+s}^{T}=\Delta m_{a}^{N}+\Delta m_{s}^{N} \end{array}$$

where *N* refers to numerically defined.

#### 2.1.2. Joints with Aluminum Substrates

In the joints with aluminum substrates, the mass gain of the adhesive can also be numerically simulated. However, the mass gain of the aluminum needs to be determined. For that, bulk aluminum plates were immersed in water and their mass gain as a function of the exposed area was established. Then, for each joint:

(4) $$\begin{array}{c} \Delta m_{s}=\frac{\Delta m_{p}}{A_{p}}\cdot A_{j} \end{array}$$

where $\Delta m_{p}$ is the mass gain of the aluminum plate and $A_{p}$ and $A_{j}$ are the exposed area of aluminum in a plate and joint, respectively. Therefore:

(5) $$\begin{array}{c} \Delta m_{a+s}^{T}=\Delta m_{a}^{N}+\frac{\Delta m_{p}}{A_{p}}\cdot A_{j} \end{array}$$

#### 2.1.3. Procedure Established for the Study of the Interphase

In this section the research methodology that was followed in this work was discussed. The objective was isolating the water uptake behavior of the interphase, which is the water uptake of the joint minus the water uptake of the adhesive and substrate. The adhesive is always a polymeric silicone material, with a known water uptake behavior; therefore, the water uptake of the adhesive was established from numerical modelling, taking into consideration the geometric constraints of each joint. The substrates were 6082-T6 aluminum, which oxidizes when in contact with water and has a mass increase due to the surfacial oxide layer; and PBT-GF30 which, as the adhesive, absorbs water and can be numerically simulated.

### 2.2. Materials

The materials chosen are used in a housing for electronic components currently used in the automotive industry. The PBT-GF30 housing is manufactured in two parts and bonded using a silicone adhesive. The housing is also bonded to an aluminum heat sink using the same silicone. Therefore, in this study, one adhesive and two different substrates were used. The adhesive was a two-component commercially available silicone. The two components were mixed 1:1 by weight and the mixture was cured at 120 °C for 25 min. The substrates were PBT-GF30 and 6082-T6 aluminum.

### 2.3. Specimen Manufacturing

#### 2.3.1. Rectangular Joints (RJ)

**Figure 3:**
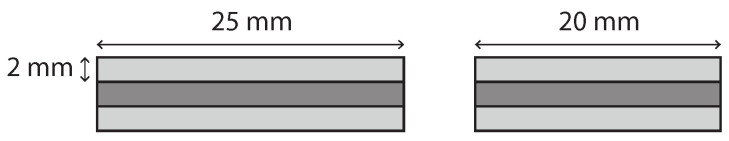
Rectangular joints manufactured.

**Figure 4:**
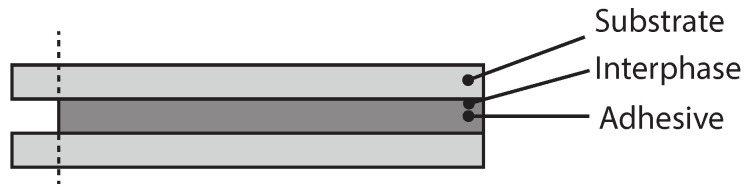
Trim RJs to ensure the adhesive is in the same plane of the end of the substrate for every side.

Joints with an overlap of 25 × 20 mm were used for the gravimetric study, Figure 3. PBT-GF30/silicone and aluminum/silicone joints were prepared and the substrate thickness was always 2 mm. To ensure the adhesive thickness, calibrated tape with the desired size was used. The joints were positioned in a mold to ensure the placement of the substrates and calibrated tape and the mold was introduced in the hot-plate press at 120 °C and 20 bar for 25 min. After curing, the excess substrate, used to place the calibrated tape, was cut, to ensure in all sides of the joint the adhesive are directly exposed to water and there is no water between substrates, without adhesive, as shown in Figure 4.

#### 2.3.2. Single Lap Joints (SLJ)

Two groups or SLJs were prepared: PBT-GF30/silicone and aluminum/silicone. For both groups, joints with 0.5, 1, and 2 mm of thickness were used. For all of the joints, the overlap was kept at 25 mm, with 25 mm width and the substrate thickness was 2 mm. The joints were placed in a mold [40] and the adhesive thickness was ensured using packing shims. The adhesive was then carefully applied to the substrates and the final joints were placed in the hot-plate press at 120 °C and 20 bar for 25 min.

**Figure 5:**
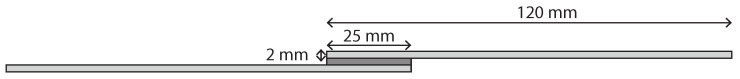
Single lap joints manufactured.

The geometry of the joints used is based in ASTM D1002 [41] and ISO 4587:1995 [42] standards, Figure 5.

### 2.4. Testing Procedures

#### 2.4.1. Gravimetric Study

The gravimetric study was performed using the RJs. The joints were fully dried in silica at 70 °C for two weeks. After drying, the joints were measured, in terms of geometry and mass, and placed in a container with distilled water at 70 °C. When in water, it was confirmed that none of the joint surfaces was in contact with the container walls or other joints. The temperature of immersion is controlled by placing the container in a climatic chamber (Memmert GmbH, Büchenbach, Germany). Periodically, the joints were removed from the immersion conditions their surfaces were dried using a paper towel, to remove water at the surface, that is not absorbed by the material, and their mass was measured. All mass measurements were performed with a microbalance with 0.1 mg of accuracy (KernToledo, Balingen, Germany). For each substrate material and each adhesive thickness at least four specimens were used.

The water uptake recorded from these joints refers to the total mass of water that ingresses the joint during immersion, either through the adhesive, through the interphase or through the pores and cracks of the adhesive. However, superficial changes of the aluminum were observed during water immersion. To understand if that phenomenon had an effect on the mass gain of the joint, three plates of aluminum were also dried in silica and immersed in water. Afterwards, their mass periodically recorded. The dimensions of the aluminum samples were measured prior to water immersion to determine their exposed area.

#### 2.4.2. Single Lap Joint (SLJ) Testing

**Figure 6:**
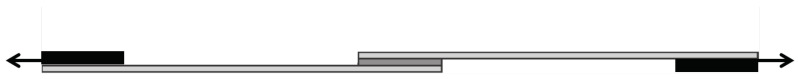
Single lap joints testing method.

The SLJs were tested after manufacturing and after being immersed in water at 70 °C for enough time for the overlap to reach a constant mass value, determined from the known behavior of the RJs. The joints were secured vertically using two grips prior to testing. To create pressure in the grips, four M6 bolts were used, with a torque of 20 Nm. To compensate for the opposing substrate and the adhesive thickness, metal tabs are positioned, Figure 6. Additionally, two center pins were used during testing to ensure proper positioning of the substrate, minimizing or preventing the specimens from slipping. The joint was tested at 2 mm/min and the load-displacement curves were recorded. The specimens tested after aging in water at 70 °C, were kept in the climatic chamber where the aging was performed at that temperature, immersed in water until testing. For each substrate material, each condition and each adhesive thickness at least three specimens were tested.

## 3. Numerical Details

Numerical models were used to simulate the water uptake behavior of joints with aluminum and PBT-GF30 substrates. For these models, a heat transfer analogy was used, where the conductivity corresponds to the coefficient of diffusion and the temperature of the nodes exposed to water corresponds to the water uptake at saturation, the other nodal temperatures are set to zero in the beginning of the model. Afterwards, the humidity enters the material following a thermal conductivity law, as the temperature is conduced from the outer nodes to the zero-temperature nodes. Similar numerical models were used by other authors with good results [4,30,31]. All the numerical models described were conducted in Abaqus^®^.

### 3.1. Aluminum Rectangular Joints

**Figure 7:**
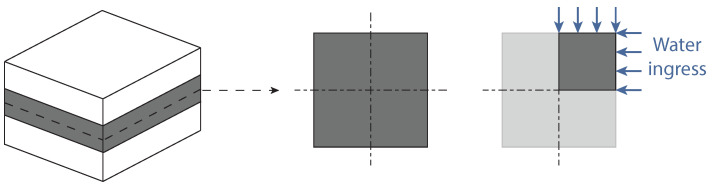
Numerical model developed for the gravimetric study of RJs with aluminum substrates, considering only water uptake through the adhesive.

This numerical model considers the ideal situation, where no water ingresses through the interphase. Therefore, each slice of adhesive parallel to the substrates has the same length exposed to water. For that reason, a 2D model, considering one of these slices, can be used to simulate the water uptake of the material, Figure 7. Since the model is symmetric in two axis, only one quarter of the slice of adhesive has to be simulated. A square mesh with a size length of 0.5 mm was used, with 4-node linear heat transfer DC2D4 elements. The temperature of each node was set to zero and the temperature of the edge nodes with arrows in Figure 7 were set to the silicone water uptake at saturation.

The side length of the simulation was first set to the exact measurements of each specimen manufactured, to understand if the differences in water uptake were significant. Afterwards, seeing the difference was negligible, the average size was used.

### 3.2. PBT-GF30 Rectangular Joints

**Figure 8:**
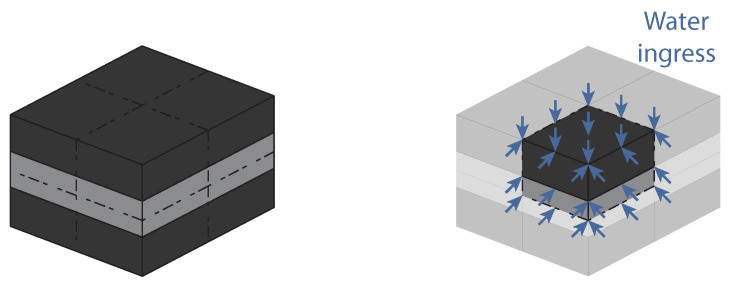
Numerical model developed for the gravimetric study of RJs with PBT-GF30 substrates, considering water uptake through the adhesive and substrate.

The numerical simulation of the RJs with PBT-GF30 substrates has to be a 3D model, since it is important to consider the water ingressing through the substrates and through the adhesive. It is also important to consider the contact between substrate and adhesive. Taking advantage of symmetry, only one eighth of the joint has to be simulated, as shown in Figure 8. For the model, a brick mesh with a size length of 0.5 mm was used, with 8-node linear heat transfer DC3D8 elements. The temperature of each node was set to zero and the temperature of the outer surface nodes with arrows in Figure 8 were set to the silicone water uptake at saturation.

### 3.3. Interface Simulation

**Figure 9:**
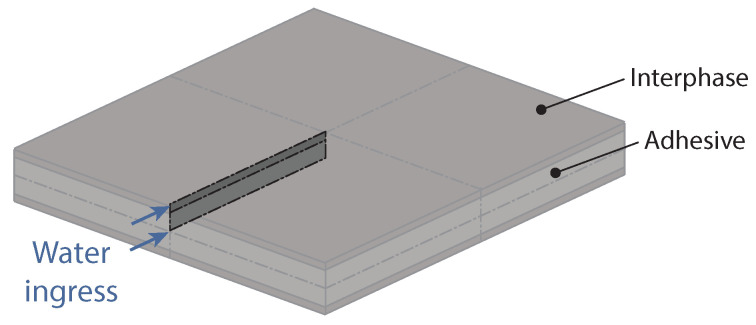
Numerical model developed to understand the water uptake of the interphase.

After the effect of the interphase was isolated, a numerical model was prepared to understand the diffusion behavior of this region of the adhesive joint. For that, since at this point the diffusion known was of the combination of adhesive and interphase, a numerical model considering the two regions was prepared. This model was a two-dimensional slice, since the diffusion at interphase was much quicker than that of the bulk adhesive and the main path of water diffusion became through the thickness, as shown in Figure 9. A maximum water uptake (temperature of the edge with arrows in Figure 9) and a coefficient of diffusion (thermal conductivity) were attributed to the interphase to fit the experimental results. To understand if the interphase thickness would have a preponderant role in the properties of the layer, different interphase thicknesses, between 0.4 and 40 μm were analyzed numerically.

## 4. Results and Discussion

### 4.1. Numerical Results

**Figure 10:**
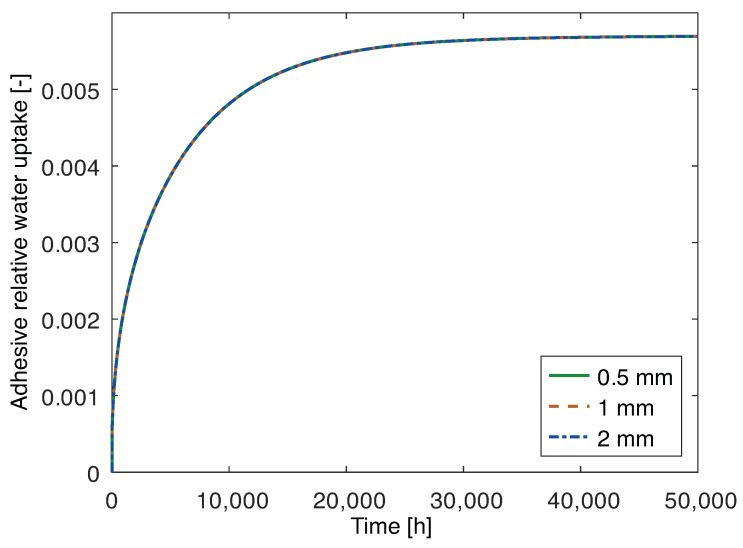
Numerical water uptake only through the adhesive layer of joints with 2 mm thick aluminum substrates and 0.5 mm, 1 mm, and 2 mm thick adhesive.

The numerical results in terms of water uptake as a function of time for the joints with aluminum substrates are shown in Figure 10. It is noticeable that the behavior is very similar, regardless of adhesive thickness, since it describes a hypothetical scenario where water only ingresses through the adhesive. In that scenario, the mass of water is proportional to the mass of adhesive, which is proportional to the adhesive thickness. Therefore, the relative water uptake, comparing to the initial mass at a given time *t*, which is the adimensional, is independent of the thickness, since it is determined from:

(6) $$M_{t}=\frac{m_{t}}{m_{0}}$$

where $m_{t}$ is the mass at time *t* and $m_{0}$ is the initial mass.

**Figure 11:**
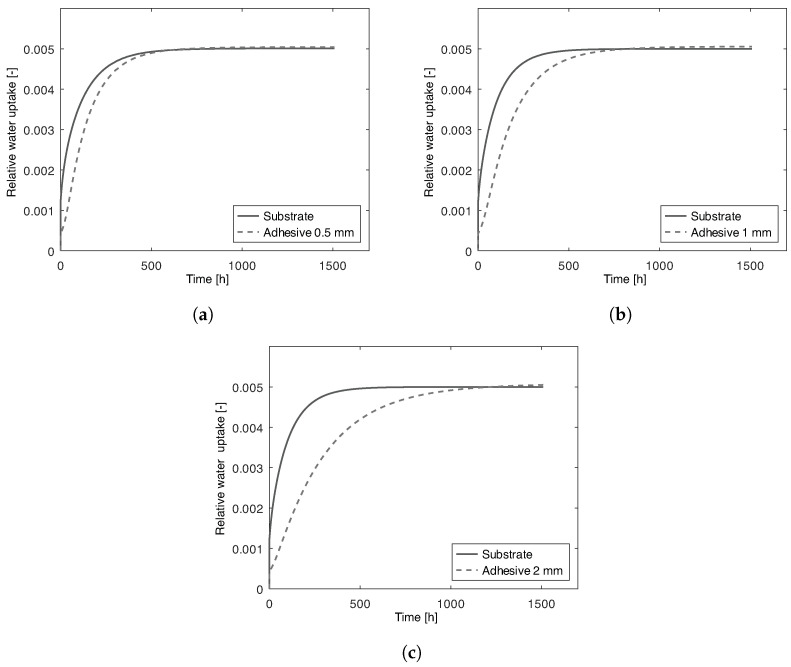
Numerical water uptake of adhesive and substrate of joints with 2 mm thick PBT-GF30 substrates and 0.5 mm (**a**), 1 mm (**b**), and 2 mm (**c**) thick adhesive.

The joints with PBT-GF30 substrates have to be analyzed using a different approach since, in this case, water flows through the substrate and the adhesive. Therefore, in Figure 11a–c, the water uptake of the substrates and adhesive for joints with adhesive thickness of 0.5 mm, 1 mm, and 2 mm, respectively, are presented.

**Figure 12:**
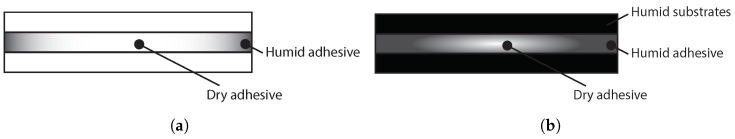
Water uptake of RJs with aluminum (**a**), and PBT-GF30 (**b**) substrates as expected from the numerical model.

The chemical and geometrical properties of the PBT-GF30 substrate are the same for all the models, i.e., the area exposed to water, substrate thickness and water uptake behavior are constant. Therefore, as expected, the water uptake behavior of the substrates is the same for all of the PBT-GF30 joints. The main difference concerns the adhesive. As seen in the aluminum joints, in Figure 12a, if water ingresses only through the adhesive, the water uptake is independent of the adhesive thickness. However, in the case of PBT-GF30, the substrate can also absorb water and, since the smaller water diffusion path is perpendicular to the adhesive/substrate interface, the substrate has a preponderant role in the water uptake of the adhesive, as shown in Figure 12b. Additionally, for this motive the theoretical water uptake of the joint with PBT-GF30 substrates is going to be significantly quicker, when compared to the water uptake of the adhesive in the joint with aluminum substrates, as it can be confirmed by the time scales in Figure 10 and Figure 11.

**Figure 13:**
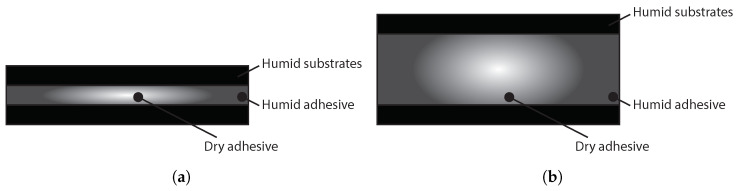
Water uptake of PBT-GF30 RJs with a thin layer of adhesive (**a**), and a thick layer of adhesive (**b**) as expected from the numerical model.

That is also the motive behind the adhesive in the 0.5 mm joint having a higher water uptake, for the same time, than the 1 mm or 2 mm joints. Since water is also absorbed through the substrate, thicker joints have a lower portion of humid adhesive at a given time, as shown in Figure 13. However, the value at saturation is the same, since the materials used are the same and water uptake is given by Equation (6).

### 4.2. Experimental Results

#### 4.2.1. Gravimetric Tests

**Aluminum rectangular joints**

**Figure 14:**
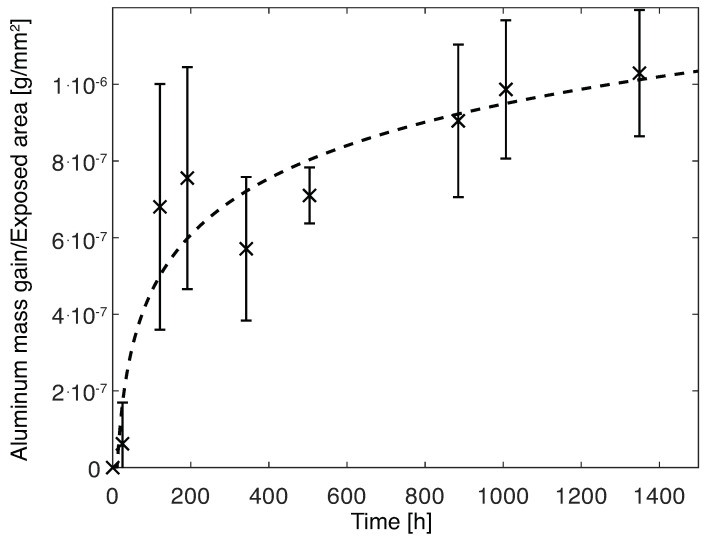
Mass gain of aluminum substrates immersed in water.

The mass gain of the aluminum substrates was determined and divided, for each time increment, by the surface area of the aluminum exposed to water. Therefore, the mass gain per mm^2^ was determined, as shown in Figure 14.

With the data collected, it is clear that a trend can be established in the time period considered, a logarithmic relation was used:

(7) $$\Delta m/A_{p}\;[g/\mathrm{mm}{}^{2}]=2.12\cdot 10^{-7}\ln t-5.16\cdot 10^{-7}$$

**Figure 15:**
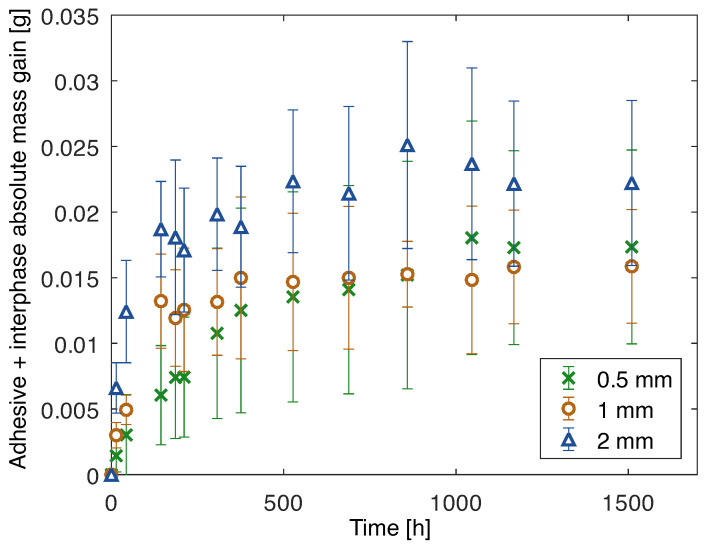
Mass gain due to water uptake of adhesive and interphase of joints with aluminum substrates and 0.5 mm, 1 mm, and 2 mm thick adhesive.

The area of aluminum exposed to water was known for each joint. Therefore, the mass increase attributed to the oxidized layer was determined and subtracted from the mass gain of the joint. Finally, it was possible to determine the absolute water mass gain of the adhesive and interphase, as shown in Figure 15.

**PBT-GF30 rectangular joints**

**Figure 16:**
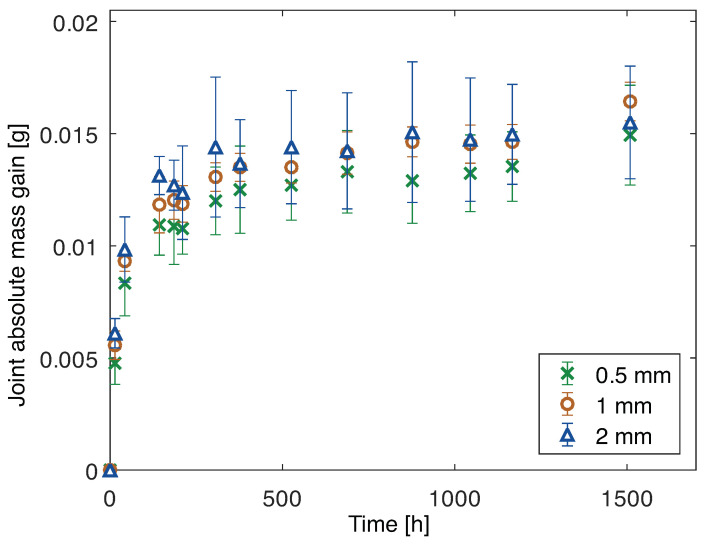
Mass gain due to water uptake of joints (adhesive, substrate and interphase) with PBT-GF30 substrates and 0.5 mm, 1 mm, and 2 mm thick adhesive.

The mass gain, through the water uptake, of the PBT-GF30/silicone joints for all of the adhesive thicknesses analyzed is shown in Figure 16.

It can be noticed that, when subjected to the same immersion conditions, the mass gain of the joints with PBT-GF30 (considering adhesive, substrate, and interphase) is similar to that of the joints with aluminum (considering adhesive and interphase). Therefore, the interphase of the aluminum joints must be absorbing an amount of water similar to that of the substrate and interphase of the PBT-GF30 joints. Additionally, at this point, the time of water absorption until a constant mass gain value was reached is approximately the same and, for the theoretical model it was significantly higher for the joints with aluminum substrates. Therefore, the water uptake of the adhesive and interphase for the joints with aluminum substrates was higher and quicker than predicted.

#### 4.2.2. Single Lap Joint Tests

**Aluminum single lap joints**

**Figure 17:**
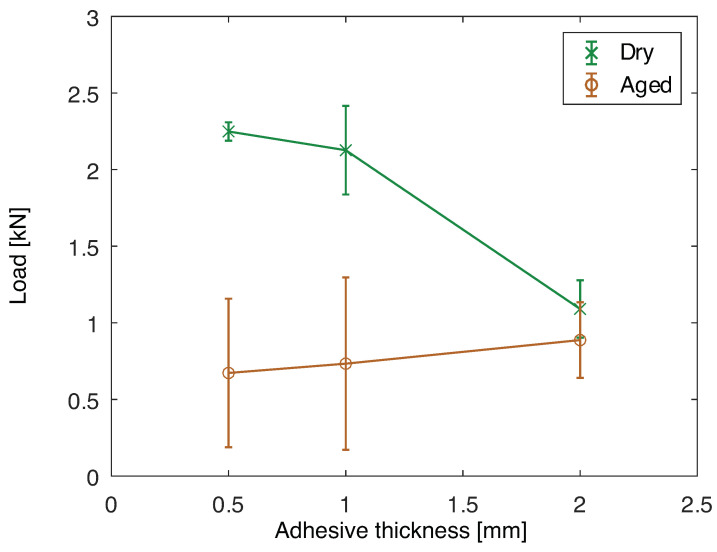
Maximum load for single lap joint tests for joints with aluminum substrates as a function of the adhesive thickness, dry and after aging at 70 °C for two months.

The maximum load of joints with aluminum substrates before and after aging is shown in Figure 17. In the dry condition, it can be seen that, between 0.5 and 1 mm of adhesive thickness the maximum load is similar. However, for 2 mm of thickness the load capacity decreases. This is primarily due to the increase in peel loading and bending moment at the overlap ends due to the higher missalignment of the substrates. Additionally, it has been seen that there is an optimum adhesive thickness to obtain maximum load capacity, and the joint with 2 mm is further away from that value [43].

**Figure 18:**
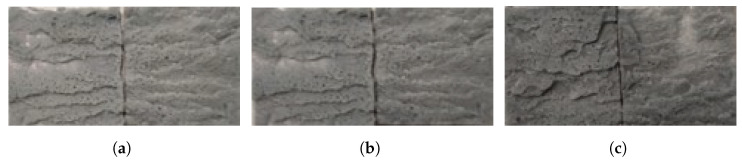
Fracture surfaces of dry joints with aluminum substrates with 0.5 mm (**a**), 1 mm (**b**), and 2 mm (**c**) of adhesive thickness.

**Figure 19:**
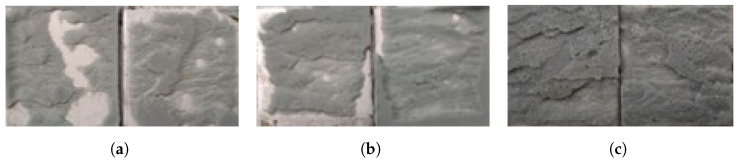
Fracture surfaces of aged joints with aluminum substrates with 0.5 mm (**a**), 1 mm (**b**), and 2 mm (**c**) of adhesive thickness.

Comparing the dry and aged conditions, it is clear that the joint load capacity significantly decreases for 0.5 and 1 mm of adhesive thickness. However, for 2 mm the load capacity is similar, which can be correlated to the fracture surfaces. The fracture surfaces of the joints when dry are presented in Figure 18 and when aged in Figure 19. The joint with 0.5 mm of adhesive thickness changes from a saw-like cohesive fracture surface, typical of silicones (Figure 18a), to a failure surface with some areas of adhesive failure (Figure 19a). The same can be seen for the 1 mm thick adhesive joints when dry and aged, Figure 18b and Figure 19b, respectively. For the 2 mm thick joints, the failure remains cohesive from the dry to the aged condition, as shown in Figure 18c and Figure 19c, respectively, which is going to be discussed in further detail in the *Discussion* section.

**PBT-GF30 single lap joints**

**Figure 20:**
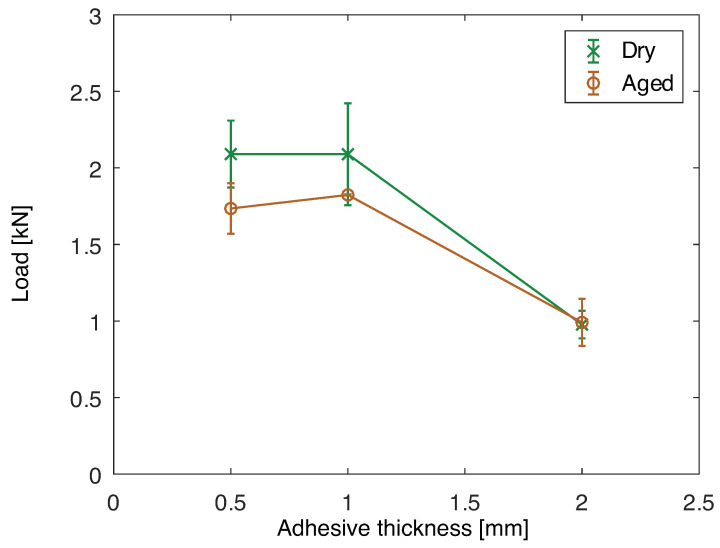
Maximum load for single lap joint tests for joints with PBT-GF30 substrates as a function of the adhesive thickness, dry and after aging at 70 °C for two months.

The maximum loads recorded for the SLJs with PBT-GF30 substrates when dry and aged are presented in Figure 20. The same trend described for the dry joints with aluminum substrates is found here. However, there is not a significant change between the dry and aged condition for these joints, with decrease in mechanical properties of the materials due to water immersion at high temperature.

**Figure 21:**
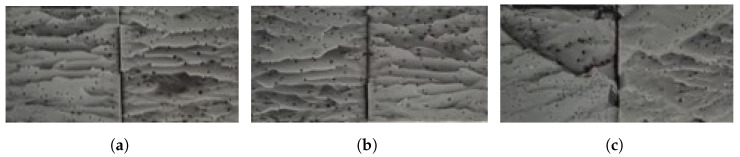
Fracture surfaces of dry joints with PBT-GF30 substrates with 0.5 mm (**a**), 1 mm (**b**), and 2 mm (**c**) of adhesive thickness.

**Figure 22:**
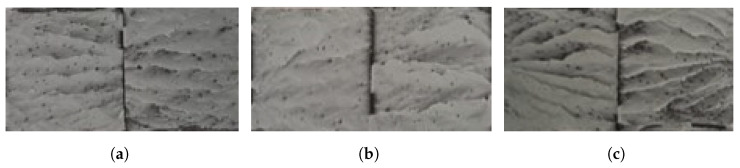
Fracture surfaces of aged joints with PBT-GF30 substrates with 0.5 mm (**a**), 1 mm (**b**), and 2 mm (**c**) of adhesive thickness.

The same similarity between the failure load for the dry and aged condition is also shown in the fracture surfaces. Both in the dry and aged conditions (Figure 21 and Figure 22, respectively), failure of the joints is cohesive through the adhesive.

### 4.3. Discussion

Joints with aluminum substrates, as seen, absorb water through the adhesive but the presence of additional diffusion paths at the interphase has been suggested. To isolate the effect of the interphase, the mass gain of the substrate was eliminated and the effect of the adhesive layer must also be disregarded.

The theoretical water uptake behavior of the adhesive is known from the numerical model, however, this water uptake was determined as a function of the mass of the adhesive from Equation (6). Therefore, for each joint analyzed, the water uptake of the adhesive must be multiplied by its initial mass to obtain the mass gain the adhesive theoretically experiences. The initial mass of adhesive can be determined from the initial mass of the joint, eliminating the initial mass of the aluminum:

(8) $$m_{a0}=m_{j0}-m_{s0}=m_{j0}-\rho_{s0}\cdot V_{s0}$$

where $m_{x0}$, $\rho_{x0}$, and $V_{x0}$ refers to the initial mass, density and volume of *x*, respectively, and *a*, *j*, and *s* to the adhesive, joint, and substrate.

**Figure 23:**
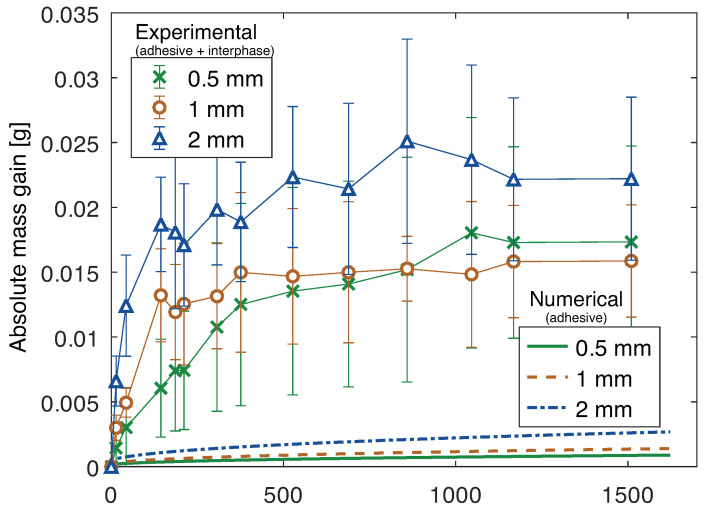
Mass gain due to water uptake of joints with aluminum substrates and 0.5 mm, 1 mm, and 2 mm thick adhesive: numerical (considering only water uptake in the adhesive) and experimental (considering adhesive and interphase).

Therefore, knowing the volume of aluminum in each joint, its density needs to be determined. This was achieved, from the initial dimensions and mass of the bulk aluminum plates, prior to water immersion, and a value of 2564 ± 26 kg/m^3^ was obtained. With this, the initial mass of adhesive was determined for each joint and its theoretical mass gain, numerically determined, was established, as shown in Figure 23.

From the numerical model of the interphase and adhesive, fitted to the experimental results, it was concluded that the water diffusion of the interphase changed between 1·10${}^{-4}$ mm^2^/s and 5·10${}^{-5}$ mm^2^/s, regardless of the thickness considered for the interphase layer, compared to 9·10${}^{-7}$ mm^2^/s of the adhesive. The main difference that must be considered, when taking into account different interphase thicknesses, is the water uptake at saturation, which must be changed since it corresponds to the mass of water divided by the mass of interphase and, if the thickness of the interphase is, for instance, half, the mass of the interphase is also half, which makes the water uptake at saturation double.

**Figure 24:**
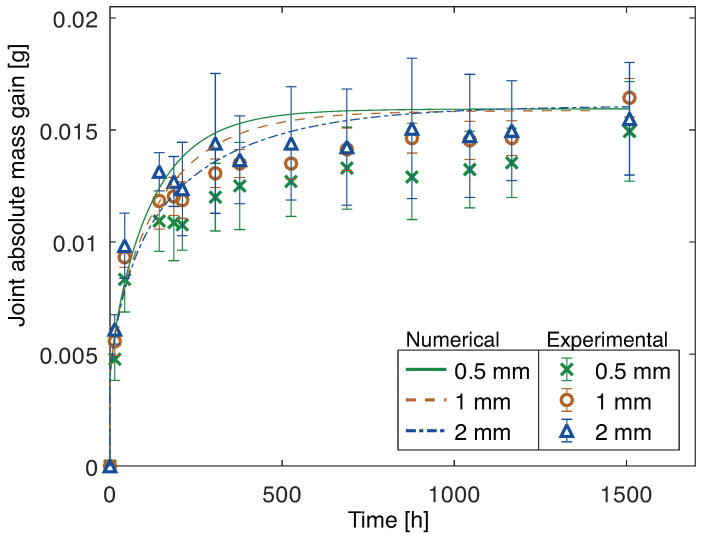
Mass gain due to water uptake of joints with PBT-GF30 substrates and 0.5 mm, 1 mm, and 2 mm thick adhesive: numerical (considering only water uptake through the adhesive and substrate) and experimental (considering adhesive, substrate, and interphase).

It could be argued that additional voids in the adhesive in the joint, when compared to the bulk plate of material, can also contribute to additional water uptake. However, this effect is negligible, as it can be confirmed from the matching results between the theoretical numerical model of the adhesive and substrate of the joints with PBT-GF30 substrate and its experimental results, as shown in Figure 24. The adhesive layer, except interphase, is expected to have the same behavior in both groups of joints, and, therefore, if the difference was due to the voids in the adhesive, that would also be clear in the joints with PBT-GF30 substrates.

Therefore, the difference in mass gain can be attributed to the differences in the interphase, which contributes to the joints with aluminum absorbing water through this region and the joints with PBT-GF30 only significantly absorbing water through the bulk materials.

The agreement between experimental (adhesive, substrate and interphase) and numerical (adhesive and substrate) results of the joints with PBT-GF30 substrates shows that water is only significantly absorbed by the adhesive and substrate. It can not be excluded that the interphase of the PBT-GF30/silicone joints can also absorb a smaller amount of water. However, that effect is not clear in our tests. This is also confirmed from the fracture surfaces. The joints with aluminum substrates exhibit adhesive failure when aged, due to the water that ingresses between the adhesive and substrate and contributes to debonding. However, the joints with PBT-GF30 substrates consistently exhibit cohesive failure through the adhesive.

It has been mentioned that water uptake can occur non negligibly through the interface or interphase for aluminum/silicone joints. The failure path, which appears to be exactly between adhesive and substrate, exhibiting adhesive failure, points to a water ingress exactly through the interface plane. However, since in a micro or nanoscale, it is very complex to affirm that the failure path is exactly between adhesive and substrate or if it is globally within the interphase, both possibilities must be considered. Additionally, since, the water uptake can be attributed more generally to the interphase. Nonetheless, these results prove that the region between adhesive and substrate must not be neglected.

These results corroborate the conclusion of Drain et al. [37], showing that, generally, the identification of the interface between composite materials with polymeric matrices and adhesive is more challenging, when compared to metal/adhesive interfaces, since the polymer of the adhesive dissolves in the polymer of the substrate and vice versa. This dissolution can be behind the apparently stronger, and more resistant to the humid environment, interphase between PBT-GF30 and silicone, when compared with the aluminum/silicone interphase.

Comparing the mass gain of the joints with PBT-GF30 substrates, as shown in Figure 24, it can be seen that, from the numerical model, the joints are expected to absorb water slower as the adhesive thickness increases. However, because the joint with 2 mm has more voids than that with 0.5 mm or 1 mm, it is seen that the water uptake at saturation is slightly higher.

When comparing between joints with aluminum substrates and different adhesive thicknesses, since the water uptake of the adhesive and the mass gain of the substrate were eliminated, the excess mass gain, which is attributed to the interphase, should be the same regardless of the adhesive thickness. However, this is not the case, which can be attributed to the slightly higher void content of the joints with 2 mm, which contributes to a higher mass gain of water.

## 5. Conclusions

The main conclusions drawn from this work are that:

- The water uptake of joints with PBT-GF30 substrates and silicone adhesive can be approximated by the numerical model that considers only the water uptake through the bulk adhesive and substrate, since the effect of the interphase is negligible;
- For the determination of the water gain of joints with aluminum substrates and silicone adhesive, the effect of the interphase must be considered, since it contributes to a significantly higher mass gain, with a coefficient of diffusion significantly higher than that of the adhesive;
- PBT-GF30/silicone adhesive SLJs exhibit cohesive failure before and after aging;
- Joints with aluminum substrates exhibit adhesive failure after aging because of the water uptake through the interphase;
- The environmental resistance of the PBT-GF30/silicone interphase appears to be higher than that of the aluminum/silicone interphase since the water absorbed through the interphase can not be identified in the gravimetric tests and after aging at 70 °C for two months failure is still cohesive in the adhesive, and for the aluminum/silicone joints adhesive failure is recorded.
